# The uncertain consequences of transferring bacterial strains between laboratories - *rpoS *instability as an example

**DOI:** 10.1186/1471-2180-11-248

**Published:** 2011-11-08

**Authors:** Beny Spira, Rodrigo de Almeida Toledo, Ram P Maharjan, Thomas Ferenci

**Affiliations:** 1 Departamento de Microbiologia, Instituto de Ciências Biomédicas, Universidade de São Paulo, Av. Prof. Lineu Prestes 1374, São Paulo-SP, Brazil; 2 Endocrine Genetics Unit (LIM-25), Endocrinology, University of São Paulo School of Medicine, SP, Brazil; 3 School of Molecular and Microbial Biosciences G08, The University of Sydney, NSW 2006, Australia

## Abstract

**Background:**

Microbiological studies frequently involve exchanges of strains between laboratories and/or stock centers. The integrity of exchanged strains is vital for archival reasons and to ensure reproducible experimental results. For at least 50 years, one of the most common means of shipping bacteria was by inoculating bacterial samples in agar stabs. Long-term cultures in stabs exhibit genetic instabilities and one common instability is in *rpoS*. The sigma factor RpoS accumulates in response to several stresses and in the stationary phase. One consequence of RpoS accumulation is the competition with the vegetative sigma factor *σ*^70^. Under nutrient limiting conditions mutations in *rpoS *or in genes that regulate its expression tend to accumulate. Here, we investigate whether short-term storage and mailing of cultures in stabs results in genetic heterogeneity.

**Results:**

We found that samples of the *E. coli *K-12 strain MC4100TF exchanged on three separate occasions by mail between our laboratories became heterogeneous. Reconstruction studies indicated that LB-stabs exhibited mutations previously found in GASP studies in stationary phase LB broth. At least 40% of reconstructed stocks and an equivalent proportion of actually mailed stock contained these mutations. Mutants with low RpoS levels emerged within 7 days of incubation in the stabs. Sequence analysis of ten of these segregants revealed that they harboured each of three different *rpoS *mutations. These mutants displayed the classical phenotypes of bacteria lacking *rpoS*. The genetic stability of MC4100TF was also tested in filter disks embedded in glycerol. Under these conditions, GASP mutants emerge only after a 3-week period. We also confirm that the intrinsic high RpoS level in MC4100TF is mainly due to the presence of an IS*1 *insertion in *rssB*.

**Conclusions:**

Given that many *E. coli *strains contain high RpoS levels similar to MC4100TF, the integrity of such strains during transfers and storage is questionable. Variations in important collections may be due to storage-transfer related issues. These results raise important questions on the integrity of bacterial archives and transferred strains, explain variation like in the ECOR collection between laboratories and indicate a need for the development of better methods of strain transfer.

## Background

Well-resourced culture collections distribute bacteria mostly as freeze-dried ampoules [1,2]. On the other hand, most research labs generally do not exchange lyophilized cultures and over the past 50 years a good proportion of bacterial exchanges were either in agar stabs or on impregnated glycerolized discs, as also used by the Coli Genetic Stock Center (CGSC). Generally, comparison of storage and shipping conditions test for viability and all of the above methods work well in this regard for *Escherichia coli*. Recently however, we became concerned about heterogeneity arising during storage and exchange of cultures for two reasons. Firstly, our recent studies with the ECOR collection [3] indicated a number of phenotypes had changed from those reported earlier (unpublished results). Others have also noted discrepancies in results with the ECOR collection between laboratories [4]. Secondly, in recently exchanged stock cultures of *E. coli *K-12 between the Ferenci and Spira laboratories, we noted heterogeneities in some of the phenotypes we routinely assay. In this communication, we investigated the source of this heterogeneity and the role of storage conditions during shippage.

The instability of cultures and possible heterogeneities have been noted in several settings. Bacteria in long term stab cultures were found to change in a number of respects [5-8]. Extended incubation over several days in rich media also gives rise to population heterogeneity [9]. In both cases, one of the targets of change was the *rpoS *gene. The sigma factor RpoS is the master regulator of the general stress response in *E. coli *[10]. RpoS coordinates the transcription of genes associated with the protection of bacteria against different types of stress, such as high osmolarity, oxygen free radicals, low temperature and others [10,11]. Bacteria that lack RpoS are more sensitive to environmental stresses, thus though *rpoS *is not an essential gene, its presence strongly increases bacteria survival rates in stressful environments.

RpoS levels are also shifted up under nutritional stress, namely carbon and phosphate starvation [12]. In stationary phase or in nutrient-limited chemostats, the accumulation of RpoS in the cytosol reduces the expression of growth-related genes due to the competition between RpoS and the vegetative sigma factor *σ*^70 ^for a limited amount of RNA polymerase core units [13]. This characterizes a trade-off in which the bacterium sacrifices growth in favour of expressing protection-related genes. Under prolonged starvation periods a genetic adjustment follows when mutations in *rpoS *or in genes that control *rpoS *expression occur, resetting the SPANC (Self Preservation and Nutritional Competence) balance [14]. The *rpoS *gene is highly polymorphic and many different alleles are found in both natural isolates and laboratory strains of *E. coli *[15-18]. This strong variation is expected given the pivotal role of RpoS in determining the SPANC balance [14] and is central to the instabilities we observe in mailed cultures.

The strain we exchanged was a derivative of MC4100, a widely used *E. coli *strain spread in many laboratories around the world. MC4100 stored at Ferenci's laboratory in Australia [19] was shown to express high levels of both RpoS and ppGpp [17,20]. This version of MC4100 (hereafter called MC4100TF) efficiently exhibits protection-related phenotypes, such as resistance to stresses and glycogen production but is less competent in metabolising alternative carbon sources. It also tends to accumulate mutations in *rpoS *following 2-3 days of growth in a chemostat under carbon or phosphate limitation [17,18]. It has been shown that a pair of point mutations at the N-terminus of the ppGpp-hydrolase SpoT is responsible for the high levels of ppGpp displayed by MC4100TF [20]. Because ppGpp has a positive effect on RpoS [21], the high level of ppGpp partially explains the strong RpoS-related phenotypes in MC4100TF. In addition, genome sequencing of this strain revealed the presence of an IS*1 *insertion in the *rssB *locus [19]. RssB acts as a chaperone that presents RpoS to the protease ClpXP, enhancing RpoS proteolyis [22]. Thus, it was postulated that disruption of *rssB *contributes to the high-RpoS level in this strain, but no direct evidence has been presented.

Here we show that MC4100 readily accumulates mutations that suppress the high-RpoS phenotype when incubated in LB-stabs either during shipping or under laboratory conditions. Ten of these segregants were analysed and shown to carry null mutations in the *rpoS *ORF. It is also demonstrated that the IS*1 *insertion in *rssB *is the main factor that upregulates *rpoS *in MC4100.

## Results and Discussion

### Segregation of *rpoS *mutants in LB stabs

LB stabs of MC4100TF have been sent from Ferenci's laboratory in Australia to Spira's laboratory in Brazil by air mail on three different occasions. Upon arrival bacteria were streaked on LB agar and isolated colonies were checked for their RpoS status by iodine staining (glycogen accumulation is enhanced by RpoS [23]). MC4100TF stains darkly with iodine, but many colonies from these shipments displayed heterogeneity in iodine staining; this generally means variations in RpoS levels [17,18]. The third shipment consisted of two LB stabs, one containing MC4100TF and the other one strain BW2952 (MC4100TF carrying a *mal*::*lacZ *fusion, but otherwise identical to MC4100TF). Bacteria were removed from each stab, suspended in 0.9% NaCl, diluted and plated on LB agar and stained with iodine. The proportion of low-staining colonies in these stabs was between 29% and 61%.

**Figure 1:**
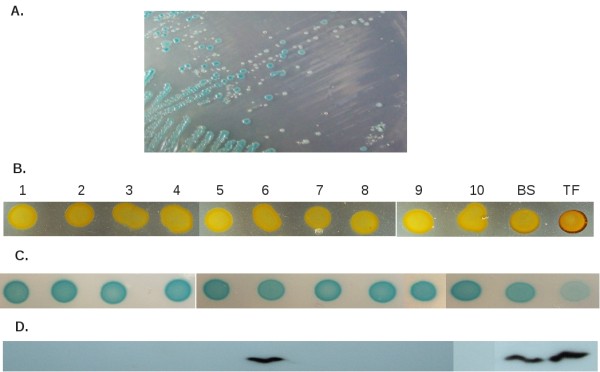
**Heterogeneity and RpoS status of MC4100TF segregants**. (A) Growth of MC4100TF colonies isolated from an LB-stab on TGP +X-P (minimal medium plate supplemented with X-P, a chromogenic substrate for alkaline phosphatase). Light-blue colonies are high-RpoS and the others with a more intense blue colour are low-RpoS segregants. Ten of these low-RpoS segregants were isolated and further analysed. Patches of overnight cultures of MC4100TF segregants (1-10), MC4100TF and MC4100BS were grown on (B) LB-agar and stained with iodine for the detection of glycogen accumulation and on (C) TGP+X-P plates. (D) Bacteria grown overnight in LB medium were assayed for RpoS by immunoblotting with monoclonal anti-RpoS antibodies. 1-10, MC4100TF segregants; BS, MC4100BS; TF, MC4100TF.

This prompted us to ask whether the shipping conditions to which the bacteria were exposed during the transcontinental flights selected mutations that caused the loss of the high-staining phenotype. To mimic the conditions during transport, a single fresh colony of MC4100TF was inoculated into an LB stab, and incubated at room temperature for 7 days. Following the incubation period bacteria were streaked on minimal medium plates supplemented with the alkaline phosphatase (AP) substrate X-P (TGP + X-P); AP expression is inversely correlated with RpoS level [18]. Several colonies were light blue, but others showed a more intense blue colour, indicating a low-RpoS status (Figure 1A). Ten of these low-RpoS segregants were isolated and further analysed.

The segregants were tested for RpoS-dependent phenotypes (iodine staining and AP basal activity), for RpoS concentration by immunoblotting and for the presence of the IS*1 *insertion in *rssB *(MC4100TF is *rssB*::IS*1*). Amplification of *rssB *by PCR showed that all segregants retained the IS*1 *insertion and were therefore, RssB deficient. Despite that, all segregants stained lightly with iodine and showed a strong blue colour on TGP+X-P plates, suggesting that RpoS is very low or lacking in these strains (Figures 1B and 1C). A western-blot analysis revealed that with the exception of segregant number 6, a band corresponding to RpoS could not be detected in the nine other strains, suggesting that they carry null mutations in *rpoS *(Figure 1D).

**Figure 2:**
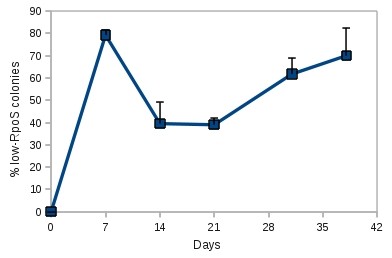
**Accumulation of low-RpoS mutants in LB-stabs**. Ten LB-stabs were inoculated with a single colony of MC4100TF and incubated at room temperature. Every week two stabs were opened, the bacteria on the top of the medium was removed, diluted and plated in duplicates. Colonies were stained with iodine and counted.

To identify the mutations present in the 10 low-RpoS segregants, the *rpoS *ORF of each strain was sequenced. The results are summarised in Table 1. Six strains (nos. 1, 2, 5, 8, 9, 10) carry an adenine deletion at position 668 of *rpoS *ORF, which results in a frameshift and the formation of premature stop codons. Segregants 3, 4 and 7 have a TAAAG deletion (Δ515-519), which also causes a frameshift. Finally, segregant 6 carries an I128N substitution in the RpoS protein. This strain displayed high levels of RpoS (Figure 2C), but behaved as an *rpoS *null mutant, suggesting that RpoS activity was severely undermined by the I128N mutation. Residue 128 is located in region 2.2 of the RpoS protein. The exact function of region 2.2 is unknown, but a tentative tertiary structure of this region showed that it is formed by a helix whose polar surface constitutes one of the primary interfaces with RNA polymerase [24]. Replacement of a hydrophobic by a polar amino acid at this position is likely to impair RpoS interaction with the core RNA polymerase, strongly inhibiting the formation of E*σ*^S ^holoenzyme and consequently the transcription of RpoS-dependent genes, such as *glgS*, involved in glycogen synthesis [23]. As predicted by the trade-off hypothesis, once RpoS loses the ability to compete with *σ*^70 ^for the binding to core RNA polymerase, the expression of *σ*^70^-dependent genes, such as *phoA *would increase, explaining the high level of AP showed by this mutant [13,17,25].

**Table 1:** Sequence analysis of low-RpoS segregants

Segregant	Change in nucleotide sequence	Change in amino acid sequence
1	Δ668A	Frameshift after aa V222

2	G343A, Δ668A	A115T, frameshift after aa V222

3	Δnt515-nt519	frameshift after aa I171

4	Δnt515-nt519	frameshift after aa I171

5	Δ668A	Frameshift after aa V222

6	T383A	I128N

7	Δnt515-nt519	frameshift after aa I171

8	Δ668A	Frameshift after aa V222

9	Δ668A	Frameshift after aa V222

10	Δ668A	Frameshift after aa V222

To further measure the frequency of emergence of *rpoS *mutations in LB stabs, a set of 15 stabs were inoculated each with a single MC4100TF fresh colony. The stabs were incubated for several weeks at room temperature and every week three stabs were tested for the *rpoS *status in each population. Each time bacteria were scraped off two different stabs, resuspended in saline, serially diluted and plated on LB agar. Bacteria from a third stab were streaked directly onto an LB plate for a qualitative analysis of the *rpoS *status. The colonies were then stained with iodine. Figure 2 shows the evolution of *rpoS *segregation in the stabs. At day 1, all tested bacteria were *rpoS*^+^, but by day 7 onwards, the presence of many low-RpoS colonies became apparent both in the quantitative (CFU count) and qualitative (streaks) plates. The exact proportion of these mutants varied from week to week, but was never lower than 40%.

A common and inexpensive alternative to LB-stabs is a bacterial suspension in filter disks in the presence of glycerol. To test this transporting method, a culture of MC4100TF was resuspended in 15% glycerol (v/v) and 0.1 ml of the suspension was applied onto a filter disk, which was placed in a small plastic bag and sealed. Glycerol filter disks were prepared along with the stabs reported in Figure 2 and stored at room temperature. Every week a pair of disks was removed from their plastic bags suspended in a small volume of saline and streaked on LB agar. Until day 21 all colonies recovered from the filter disks displayed a high-RpoS phenotype (stained dark brown with iodine). From day 31 onward a significant proportion (approx. 50%) of the bacteria recovered from the filter disks were low-RpoS. Furthermore, there was an increasing reduction in the number of colonies recovered every week, possibly due to prolonged starvation and dehydration of the filter disks (despite the sealing of the plastic bags). It is clear, though, that the glycerol filter disks preserved the genetic integrity of the bacteria for a longer period of time than the LB-stabs. Therefore, the use of glycerol filter disks for bacterial shipment is preferable.

The data presented here indicate that the use of LB-stabs for the exchange of bacteria between laboratories is undermined by genetic instability. Alternative storage and shipment forms, such as freeze-drying, glycerol filter disks or dry ice must be considered. Some of them are costly (shipment of glycerol stocks in dry ice) or dependent on specific equipments (lyophiliser) and none is free of drawbacks. As a matter of fact, induction of mutations during the freeze-drying process has been reported [26,27]. Glycerol filter disks provide an inexpensive and easy alternative for bacterial shipping. Since the filters lack essential nutrients we expect very little or no bacterial growth and hence a significant reduction in mutant segregation.

Ever since the pioneer work of the Kolter group [28], several papers have reported the occurrence of *rpoS *mutations that confer selective advantage in stationary phase (the GASP phenotype) [8,9,29]. Accordingly, sequence variation of *rpoS *in *E. coli *natural isolates is extensively well documented [3,3,16,30-32]. Even in archival cultures, a high proportion of *rpoS *mutants has been observed [8]. This is not surprising because genomic rearrangements in stab cultures stored for long periods of time are common [4-6,33], implying that long-term storage in stabs produces an environment that selects for a variety of mutations. Indeed, discrepancies among archival strain collections, like the ones found in the ECOR collection [4] and in bacterial strains used in compendial microbiological tests [34] are not uncommon.

To the best of our knowledge, this is the first report on rapid evolution in LB-stabs. This has implications not only for the storage of bacteria in the laboratory, which is less significant today because most bacteria collections are kept at -70°C freezers, but mainly to bacterial exchange by the scientific community. We demonstrated the emergence of mixed populations of *rpoS*^+ ^and *rpoS *attenuated bacteria in LB-stabs of MC4100TF (a widely used *E. coli *strain) even after one-week incubation. This strain exhibits high levels of RpoS. High-RpoS strains tend to accumulate mutations in *rpoS *in order to reset the SPANC balance, i.e., to eliminate the competition between *σ*^70 ^and RpoS enhancing the transcription of growth-related genes. However, RpoS loss is not restricted only to MC4100TF as other *E. coli *strains, even some with not particularly high RpoS (such as MG1655) have shown to accumulate mutations in *rpoS *under nutrient limiting conditions [3,17,18]. Although in the present study, the only tested variation was regarding *rpoS*, it is clear that other genes, such as *lrp *(a GASP allele of *lrp *has been isolated under prolonged starvation [35]) may also be affected during short-term incubation in LB-stabs, and this caveat should be taken into account when posting bacteria via air mail. It should also be noted that evolution in LB stabs are likely to occurr in other bacteria species, even in the ones regarded as more stable (such as Gram positive bacteria). This possibility can be empirically tested in the future.

### The relation between RssB and RpoS in MC4100 derivatives

Sequence analysis of MC4100TF showed that it carries an IS*1 *insertion at the 5'-end of the *rssB *ORF [19], whose product facilitates the proteolysis of RpoS [22,36]. Disruption of *rssB *is likely to contribute to the intrinsic high level of RpoS in MC4100TF because stocks of MC4100 maintained in other laboratories around the world do not carry the IS*1 *insertion in *rssB *and do not exhibit high levels of RpoS [19], but a direct evidence is still lacking. Furthermore, none of the tested segregants have reverted the *rssB *mutation, though a *rssB*^+ ^allele would supposedly diminish the level of RpoS in this strain.

**Figure 3:**
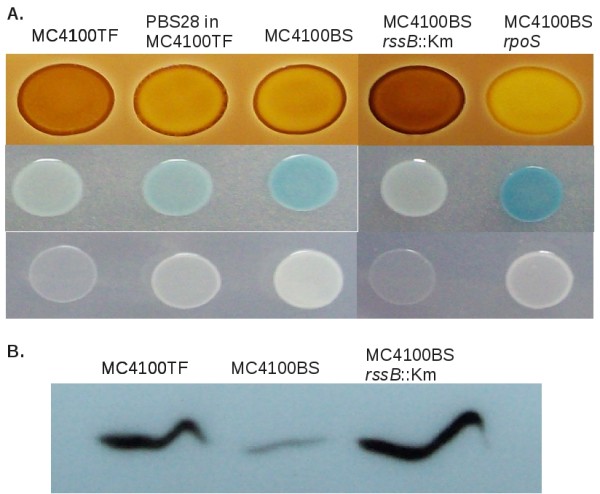
**RpoS-dependent phenotypes in MC4100 stocks and the role of *rssB***. (A) 10 *μ*l patches of strains MC4100TF, MC4100BS, MC4100BS *rssB*::Km, MC4100BS *rpoS*::Tn*10 *and MC4100TF transformed with plasmid pBS28 (p*rssB*^+^) were grown on LB-agar, TGP+X-P plates and TAP plates (minimal medium supplemented with acetate as the sole carbon source). Bacterial patches in LB-agar were stained with iodine to measure glycogen accumulation. (B) Immunoblotting of RpoS in strains MC4100TF, MC4100BS and MC4100BS *rssB*::Km. Bacteria grown overnight in LB medium were assayed for RpoS level with monoclonal anti-RpoS antibodies.

Therefore, to test if the IS*1 *insertion in *rssB *is related to the intrinsic high-RpoS level in MC4100TF, a series of experiments was conducted. The *rssAB*^+ ^operon was cloned in a low-copy plasmid (pBS28) and transformed into MC4100TF to complement the *rssB*::IS*1 *mutation. In parallel, the *rssB *gene of MC4100BS (a stock of MC4100 maintained in our laboratory, which is low-RpoS and *rssB*^+ ^[18,19]) was knocked-out by the introduction of a KmR cassete. RpoS-dependent phenotypes, such as glycogen accumulation, alkaline phosphatase activity and growth on acetate as the sole carbon source (inhibited by high RpoS) [17] were assayed in strains MC4100TF, MC4100BS, MC4100BS *rssB*::KmR and MC4100TF carrying pBS28. Figure 3A shows that MC4100TF and MC4100BS *rssB*::KmR stained dark with iodine, showed low AP activity and poor growth on acetate, as expected for high-RpoS strains. Conversely, MC4100BS and MC4100TF carrying pBS28 accumulated less glycogen, more AP and grew stronger on acetate, all characteristics of low-RpoS strains. An immunoblot with an anti-RpoS serum confirmed that both MC4100TF and MC4100BS *rssB*::KmR displayed high levels of RpoS when compared to MC4100BS (*rssB*^+^) (Figure 3B). Altogether, these results demonstrate that the intrinsic high level of RpoS in strain MC4100TF is indeed caused by the IS*1 *insertion in *rssB*. There is a priori no reason why the *rssB*^+ ^genotype would not be restored in MC4100TF, but we did not detect it in any of the tested low-RpoS segregants. On top of its role in RpoS proteolysis *rssB *helps modulating polyadenylation and degradation of specific mRNAs, and its overexpression is toxic for the cell [37]. Thus, it is possible that under the conditions tested (incubation in LB stabs), mutations in *rpoS *would be less costly than reversion of the *rssB*::IS*1 *mutation. Indeed, many evolution experiments in chemostats with MC4100TF have been carried out, but reversion of the *rssB*::IS*1 *mutation has not been observed in any of them [38,39].

Another potential input that might be upregulating *rpoS *in MC4100TF is ppGpp [20,21]. ppGpp synthesis and degradation are driven by the products of the *relA *and *spoT *genes [40,41]. MC4100TF carries two mutations in spoT (a H255Y substitution and a +QD insertion between residues 82 and 83) [20]. When transferred to another *E. coli *strain, this *spoT *allele increased both ppGpp and RpoS levels. However, high levels of ppGpp and the same *spoT *mutations are present in MC4100BS as well [20]. Therefore, ppGpp cannot explain the high RpoS level in MC4100TF, which is mainly due to the knock-out of *rssB*.

## Conclusions

Mutations in the *rpoS *locus or in genes that regulate *rpoS *expression are powerful drivers of genetic instability or adaptability both in the laboratory and in nature. Growth and storage of bacteria in LB-stabs for short periods, such as the time it takes to mail a letter between different continents, is sufficient for the accumulation of *rpoS *mutations in high proportions. Mutations that inactivate or attenuate RpoS confer on the bacteria the GASP phenotype, explaining why they are so common across the species *E. coli*. A better alternative for the shipment of bacterial strains is the use of glycerol filter disks, in which a small volume of a bacteria culture resuspended in 15% glycerol is applied to a filter disk in a sealed plastic bag. Finally, of the many inputs that regulate *rpoS*, it was demonstrated that the high level of RpoS in strain MC4100TF is mainly due to an IS*1 *insertion in *rssB*.

## Methods

### Bacterial strains, plasmids and media

The strains used in this study were MC4100 (F^- ^*ara*D139 (*argF-lac*)U169 *rpsL*150 *deoC*1 *relA1 thiA ptsF*25 *flbB*5301 *rbsR*) stored in TF and BS laboratories; KM32 (*lac *Δ(*recC ptr recB recD*)::Ptac-*gam-red cat*) that carries a chromosomal copy of the *λ*-Red recombination system [42] and DH10B (F- *mcrA *Δ(*mrr-hsdRMS-mcrBC *80d*lacZ*ΔM15 Δ*lac*X74 *endA1 recA1 deoR *(*ara leu*) 7697 *araD*39 *galU galK nupG rpsL*), used as recipent for plasmid transformation.

Plasmid pUC4K is a pUC19 derivative that carries a KmR cassete [43]. pGEM T-easy is a cloning vector (Promega). pWKS130 is a low-copy cloning vector [44]. pBS23 is a pGEM T-easy derivative that carries *rssB*^+^. pBS25 is as pBS23 except that a KmR cassete (from pUC4K) was inserted into *rssB*. pBS28 is a pWKS130 derivative that carries the *rssAB*^+ ^operon.

TGP [45] plates contained 0.2% glucose, 1 mM KH_2_PO_4 _and 40 *μ*g/ml X-P. LB plates and stabs were as described [46]. Cells were grown overnight in either LB broth or in liquid T-salts supplemented with 0.2% glucose and 1 mM KH_2_PO_4 _at 37°C.

### Bacterial storage and sampling

LB-stabs were innoculated with a single colony and immediately sealed by screwing down the tube lid. Following incubation at room temperature for different time lengths, bacteria samples were removed from the stabs either with a sterile glass rod (and subsequently streaked on a plate) or by scraping off the upper layer of the stab with a sterile metal stick.

Bacteria were then transferred to a microtube filled with 1 ml 0.9% NaCl and the turbidity of the sample was measured in a spectrophotometer. Bacteria were further diluted in 0.9% NaCl (usually 10^6 ^fold) and 0.1-0.2 ml were plated onto LB or TGP plates in duplicates. Glycerol filter disks were prepared by suspending a fresh colony in 100 *μ*l 15% glycerol, A filter disk embedded with the bacteria suspension was sealed in a plastic bag. At appropriate time intervals, the plastic bag was opened and the disk transferred to a microtube filled with 200 *μ*l 0.9% NaCl. 20 *μ*l of this suspension was applied to the surface of a LB plate and streaked.

### Determination of RpoS status

The level of RpoS was qualitatively assessed by staining intracellular glycogen with an iodine solution as described [23] or by visualising AP basal level with the chromogenic substrate X-P (5-bromo-4-chloro-3-indolyl-phosphate) [18]. For the iodine staining, patches of bacteria or diluted samples were grown overnight on LB plates, stored at 4°C for 24 h and then flooded with iodine. The intensity of the brown colour varies according to glycogen concentration in the cell and indirectly reveals the level of RpoS [17,18]. *rpoS*^+ ^strains stain brown to dark brown.

### Western-blot of RpoS

Western-blot analyses were performed essentially as described [47]. Briefly, 2 *× *10^9 ^bacteria grown overnight in LB-broth were resuspended in 200 *μ*l application buffer (0.5 M Tris/HCl, 2% SDS, 5% 2-mercaptoethanol, 10%, v/v, glycerol and 0.01% bromophenol blue) and boiled for 5 min. Proteins were resolved in a 12.5% denaturing polyacrylamide gel and transferred to a nitrocelullose membrane (GE HealthCare) by capillary action. Following blocking with 5% skim milk, the membrane was incubated with 2, 000-fold diluted monoclonal anti-RpoS antibodies (Santa Cruz) and 20, 000-fold diluted peroxidase conjugated anti-mouse IgG (Pierce). The Super Signal West Pico kit (Pierce) was used to detect the RpoS bands as recommended by the manufacturer and the membrane was exposed to X-ray films.

### Knock-out of *rssB*

A KmR cassete was inserted into *rssB *ORF by homologous recombination using the *λ*-Red system as described [48]. The *rssB *gene was PCR amplified from *E. coli *chromosome with primers rssB94F (5'-CGCACCAACATTTGACCAG) and rssB1368R (5'-GTATCGCATCCCAGTATATCAG) and ligated into pGEM T-easy (Promega), resulting in plasmid pBS23. The KmR gene was excised from pUC4K by digesting with EcoRI and ligated into the MunI site of *rssB *in pBS23. The resulting plasmid (pBS25) was used as a template for the PCR amplification of the *rssB*-KmR fragment. The PCR product was resolved by electrophoresis, extracted from the gel and purified using the Wizard SV gel and PCR clean-up system (Promega). The linear DNA carrying *rssB*-KmR was electrotransformed into strain KM32 and plated on Km plates. One out of three colonies was KmR and AmpS, suggesting that the resistance to Km was due to insertion of KmR into the chromosome and not due to transfomation of pBS25 leftovers. The KmR insertion in *rssB *was verified by PCR. The *rssB*::KmR mutation was transferred to strain MC4100BS by P1 transduction [46].

### Cloning of *rssAB*

A DNA fragment containing the entire *rssAB *operon was obtained by PCR amplification with primers rssA231F (5'-CCATCAATTCGGCACGTAAC) and rssB1368R (5'-GTATCGCATCCCAGTATATCAG) and cloned in pGEM T-easy (Promega) following the manufacturer instructions. The resulting plasmid was then digested with EcoRI and the *rssAB *fragment was ligated to the low-copy vector pWKS130 [44] previously linearised with EcoRI, resulting in plasmid pBS28. Strain DH10B was used as a recipient for DNA transformation.

### PCR and sequencing

The *rpoS *ORF from each strain was amplified by PCR using the sets of primers 429F (5'-GGAACAACAAGAAGTTAAGG)/9274R (5'-CAGACCACGATTGCCATAAC), C600F (5'-CAGGGATCCACACAGCGTGT)/9700R (5'-GTCATCTTGCGTGGTATCTTCC), 9363F (5'-CATACGCAACCTGGTGGATT)/10086R (5'-GTGTTAACGACCATTCTCGG). The PCR products were confirmed by electrophoresis in a 1.5% agarose gel and purified with the Concert Rapid PCR Purification System kit (Life Technologies, Bethesda, MD). Sequencing reactions were directly performed from purified PCR products using the same primers for both strands and Big Dye Terminator v3.1 (Life Technologies, Foster City, CA). Sequencing was carried out on an automated sequencer (ABI Prism 3130XL DNA Analyzer, Applied Biosystems, Foster City), according to the manufacturer recommendations. The *rpoS *sequences from the LB stabs isolates were deposited in the GenBank database under the accession numbers JN813535-JN813544.

## Abbreviations

AP: Alkaline Phosphatase; CFU: Colony Forming Units; CGSC: Coli Genetic Stock Center; GASP: Growth Advantage in the Stationary Phase; SPANC: Self Preservation and Nutritional Competence; X-P: 5-bromo-4-chloro-3-indolyl-phosphate.

## Authors' contributions

BS conceived and desgined the study, performed most experiments and wrote the manuscript. RAT sequenced the *rpoS *mutants. TF suggested experiments, wrote and corrected the manuscript. RPM prepared cultures for transportation. All authors have read and approved the final manuscript.
